# A Systematic Review of Human Infections by *Pseudomonas mendocina*

**DOI:** 10.3390/tropicalmed5020071

**Published:** 2020-05-03

**Authors:** Petros Ioannou, Georgios Vougiouklakis

**Affiliations:** Department of Internal Medicine & Infectious Diseases, University Hospital of Heraklion, 71500 Heraklion, Crete, PC, Greece; billahem@hotmail.com

**Keywords:** *Pseudomonas mendocina*, endocarditis, meningitis, SSTI, bacteremia

## Abstract

*Pseudomonas mendocina* is a Gram-negative, rod-shaped, aerobic bacterium that belongs in the family Pseudomonadaceae and has been isolated from water and soil. Even though it is thought to cause infections quite rarely in humans, it can cause severe infections even in immunocompetent individuals. The aim of this study was to systemically review all cases of human infection by *P. mendocina* in the literature and describe their epidemiology, microbiology, antimicrobial susceptibility, treatment and outcomes. Thus, a systematic review of PubMed for studies providing epidemiological, clinical, microbiological as well as treatment data and outcomes of *Pseudomonas mendocina* infections was conducted. In total, 12 studies, containing data of 16 patients, were included. The commonest *P. mendocina* infections were infective endocarditis, central nervous system infections and skin and soft tissue infections (SSTIs). Fever was the main presenting symptom, while sepsis was evident in almost half the patients. *Pseudomonas mendocina* was susceptible to most antibiotics tested. Mortality was low in all different infection types. Third or fourth generation cephalosporins and quinolones are the commonest agents used for treatment, irrespectively of the infection site.

## 1. Introduction

*Pseudomonas mendocina* is a Gram-negative, rod-shaped, aerobic bacterium that belongs in the family Pseudomonadaceae. It was first isolated from water and soil in Mendoza, Argentina in 1970, and was named after that [1]. Even though it is thought to cause infections quite rarely in humans, it can cause severe infections, such as infective endocarditis (IE) or central nervous system (CNS) infections, even in immunocompetent individuals [2,3]. The aim of this study was to systemically review all cases of human infection by *P. mendocina* in the literature and describe their epidemiology, microbiology, antimicrobial susceptibility, treatment and outcomes.

## 2. Methods

### 2.1. Data Search

For the conduction of this review, the Preferred Reporting Items for Systematic Reviews and Meta-Analyses (PRISMA) guidelines were adopted [4]. A search of PubMed MEDLINE with the following text-words: pseudomonas[tw] AND mendoc*[tw] was used for identification of eligible studies. Day of last search was 4 January 2020.

### 2.2. Study Selection

Studies were included in analysis if the following criteria were met: (1) published in English; (2) reporting data on clinical characteristics, microbiology, treatment and outcomes. From the analysis, studies with the following criteria were excluded: (1) secondary research studies (such as reviews), editorials and articles not reporting primary research results; (2) studies not in humans; (3) studies on colonization but not infection by *P. mendocina*; (4) studies not in English. Two investigators (PI, GV) used abstrackr [5] to independently review the titles and abstracts of the resulting references and also retrieved and rescreened the full text publications of potentially relevant articles. Study selection was based on consensus. Reference lists of the studies that were included were searched for relevant articles.

### 2.3. Study Outcomes

The primary study outcomes were to record: (a) the epidemiology and medical history of patients with *P. mendocina* infection and (b) the type of *P. mendocina* infections included in the literature. Secondary outcomes included recording: (a) clinical characteristics for different type of infections, (b) microbiological data on *P. mendocina* infections, (c) their treatment and (d) their outcomes.

### 2.4. Data Extraction and Definitions

Each eligible study was examined by two investigators (PI, GV) who also extracted the data. Extracted data included study type, country and year of publication; patient demographic data (gender and age); patient’s relevant medical history (diagnosis of prosthetic cardiac valve, autoimmune disease, cancer or chronic kidney disease); data on infection and microbiology (predisposing factors, such as neutropenia or presence of a central venous catheter (CVC), isolated bacterial strains, infection site, presence of complications); antimicrobial susceptibility data, treatment of the infection; and outcomes (i.e., death or cure). Relation of death to the infection was reported according to the authors of the study. The recorded complications included any clinical deterioration or organ dysfunction that was considered by the authors of the study to be related to the *P. mendocina* infection. The quality of evidence of the outcomes of included studies was assessed using the Grading of Recommendations Assessment, Development and Evaluation (GRADE) [6].

## 3. Results

### 3.1. Literature Search

**Figure 1:**
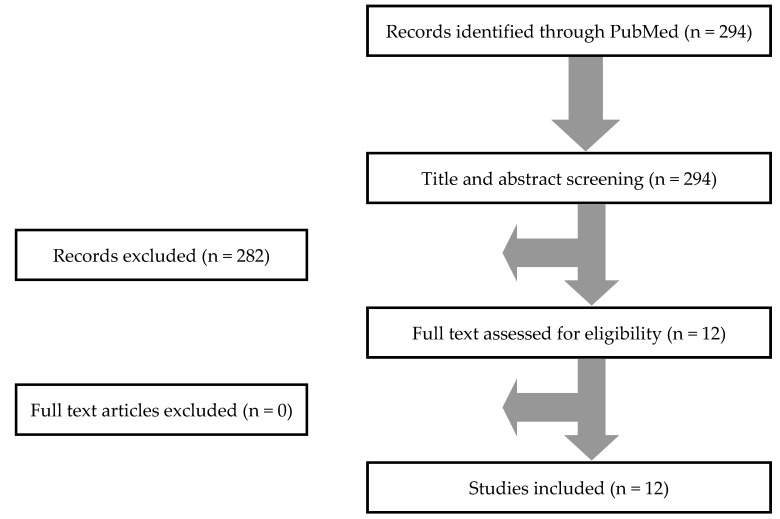
Preferred Reporting Items for Systematic Reviews and Meta-Analyses (PRISMA) flow diagram.

In total, 294 articles from PubMed were screened. After title and abstract review, 12 studies were selected for full text review. None of these studies were excluded. Hand screening of the included articles’ references did not identify any additional studies. Thus, 12 studies met the inclusion criteria of the present study [2,3,7,8,9,10,11,12,13,14,15,16]. Figure 1 shows a graphical representation of the review process.

### 3.2. Included Studies’ Characteristics

The 12 studies that were included in this analysis involved 16 patients in total. Among them, six were conducted in Asia, four in America and two in Europe. There were 10 case reports and two case series, thus, the overall quality of evidence that contributed to this systematic review was rated as low to very low [6].

### 3.3. Epidemiology, Microbiology, Treatment and Outcomes of P. mendocina Infections

Age of patients ranged from 22 to 86 years; the mean age was 53.5 years and 75% were male. The commonest infections were IE and CNS infections in 25% (four patients) each, skin and soft tissue infections (SSTIs) in 18.8% (three patients), musculoskeletal infections and bacteremia (without IE) in 12.5% (two patients) and peritoneal dialysis-associated peritonitis in 6.3% (one patient). A concomitant infection was noted in 21.4% (three out of 14 cases). Fever was present in 70% (seven out of 10 cases), and sepsis in 44.4% (four out of nine cases). Resistance to ampicillin was noted in 80% (four out of five cases with available data), to co-trimoxazole in 33.3% (one out of three cases), to carbapenems in 10% (one out of 10 cases), and to third or fourth generation cephalosporins, aminoglycosides, quinolones, piperacillin–tazobactam and colistin in 0% (0 out of 11, 10, 7, 6 and 1 case, respectively). The commonest antibiotics for the treatment of *P. mendocina* infections were third or fourth generation cephalosporins in 53.3% (eight out of 15 cases with available data), quinolones in 33.3% (five cases), aminoglycosides in 20% (three cases), carbapenems and colistin in 13.3% (two cases) each, and aminopenicillins, combination of piperacillin and tazobactam and co-trimoxazole in 6.7% (one case) each, while in 33.3% (five cases), surgical treatment was also performed. Clinical cure was achieved in 93.3% of cases (14 out of 15 patients with available data) and overall mortality was 6.7% (one patient) but the mortality attributed directly to *P. mendocina* infection was 0%. The characteristics of patients with *P. mendocina* infections are shown in Table 1.

### 3.4. Infective Endocarditis

Among the 16 patients with *P. mendocina* infections, four (25%) involved IE [3,7,8,9]. Among patients with IE, 75% were male (three out of four cases) and the mean age was 46 years. Patients with previous heart surgery represented 50% (two out of four cases); 25% had a prosthetic valve and 25% had ventricular septal defect, double-outlet right ventricle and pulmonary stenosis, while 75% of patients (three out of four cases) did not have any clear predisposing factor for developing IE. Bacteremia was present in 100% (four cases). Presence of fever was noted in 100% (four out of four patients), while 66.7% (two out of three patients with available data) were septic.

Resistance to ampicillin was 75% (three out of four cases), while resistance to aminoglycosides, third or fourth generation cephalosporins, piperacillin–tazobactam, quinolones, carbapenems, co-trimoxazole and colistin was 0%. Presence of a concomitant infection was noted in 25% (one out of four cases), namely, an SSTI by another organism. For the treatment of *P. mendocina* IE, third or fourth generation cephalosporins, quinolones and aminoglycosides were used in 50% (two out of four cases), and carbapenems and the combination of piperacillin with tazobactam were used in 25% (two cases) each. Surgery was performed in 50% (two cases): in 25% (one case) mitral valve replacement and in 25% (one case) tricuspid valve repair. Among the three patients with available data, the median duration of symptoms was seven days, with a minimum of four and a maximum of 45 and the median duration of treatment was 49 days, with a minimum of 42 and a maximum of 91. Clinical cure was achieved in 100% (four out of four cases). Overall mortality was 0% (0 patients). The characteristics of patients with *P. mendocina* IE are shown in Table 2.

### 3.5. CNS Infections

Among the 16 patients with *P. mendocina* infections, four (25%) involved CNS infections [2]. Among patients with CNS infections, 50% were male (two out of four) and the mean age was 69.5 years. Among these infections, 75% (three out of four) were hospital acquired and 25% were community acquired, while 50% (two out of four patients) did not have any clear predisposing factors for *P. mendocina* infection.

Treatment of *P. mendocina* CNS infections included third or fourth generation cephalosporins in 75% (three out of four cases), and a carbapenem was used in 25% (one case). Clinical cure was achieved in 100% (four out of four cases) and overall mortality was 0%. The characteristics of patients with *P. mendocina* CNS infections are shown in Table 3.

### 3.6. Miscellaneous Infections

Other less frequent infections by *P. mendocina* included SSTIs [12,13] in 18.8% (three out of 16 cases), bacteremia (without IE) [10,11] and musculoskeletal infections [14,15] in 12.5% (two cases) each, and peritoneal dialysis-associated peritonitis [16] in 6.3% (one case).

## 4. Discussion

*Pseudomonas aeruginosa* is among the leading causes of hospital infections, being a cause of hospital acquired pneumonia, bacteremia and infections of the urinary tract, contributing to increasing hospital costs and mortality [17]. On the other hand, non-*Pseudomonas aeruginosa* strains are much less frequently reported in the literature, with some of them being relatively unknown, such as *P. mendocina*. This study summarizes the available information on epidemiology and clinical presentation, treatment and outcomes of infections by *P. mendocina*.

Overall, IE, CNS infections and SSTIs were the commonest infections noted, and most patients were young and male. Interestingly, in almost half the cases, no predisposing factor for *P. mendocina* infection was noted. In terms of antibiotic resistance, *P. mendocina* was highly resistant to ampicillin and had low levels of resistance to co-trimoxazole and carbapenems. Otherwise, resistance to third or fourth generation cephalosporins, aminoglycosides, quinolones, piperacillin–tazobactam and colistin was zero, even though there were some missing data from some studies; thus it could be that some cases of antibiotic resistance were missed, as would one expect, for example, with the case of carbapenem resistance, where concomitant resistance to piperacillin–tazobactam would reasonably be anticipated.

Considering the abovementioned antibiotic resistance data, it is of no surprise that the commonest regimens used for the treatment of *P. mendocina* infections were third or fourth generation cephalosporins and quinolones, in more than 30% of cases each, while aminoglycosides, carbapenems, colistin, piperacillin with tazobactam, aminopenicillins and co-trimoxazole were used in less than 20% of cases. Interestingly, to our knowledge, there are no data on antimicrobial resistance of *P. mendocina*; thus, this study is the first one to systematically report antimicrobial susceptibility data of *P. mendocina*, allowing for choosing appropriate treatment for those infections.

IE is the commonest *P. mendocina* infection reported in the literature. Previous cardiac surgery and presence of a prosthetic cardiac valve were very common in patients with *P. mendocina* IE, implicating these factors as potential risks. In terms of antibiotic resistance, ampicillin resistance was very high, while no resistance to other antibiotics was noted. Thus, for treatment of *P. mendocina* IE, beta-lactamic antibiotics and quinolones were the commonest antibiotics used, in combination with an aminoglycoside in most of the cases. Surgical management was chosen in almost half of the cases, a proportion higher than that in other studies [18,19]. Mortality was zero, remaining very low as compared to that caused by other micro-organisms reported in the literature [18,19].

*P. mendocina* CNS infections were equally as common as IE in the literature. Half the patients had a hospital acquired infection, with one of them having an external ventricular drainage device. Surprisingly, 50% of patients, namely, those that had a community acquired CNS infection, did not have an obvious predisposing factor for developing meningitis from this particular micro-organism. The mainstay of treatment included third or fourth generation cephalosporins and carbapenems, while clinical cure was achieved in all the cases. Mortality was zero. This is in vast contrast with the CNS infections caused by other micro-organisms, and even more specifically from *P. aeruginosa*, that carry significant mortality [2,20,21].

This systematic review has some limitations. First of all, it consists of case series and case reports, which means that the results must be studied with caution, since case reports describe unusual manifestations, implying that the common ones could be underrepresented in such a systematic review. For example, it could be that many cases of *P. mendocina* bacteremias occur but are not published, even in immunocompetent patients, thus affecting the rank of these infections among others. However, this methodology was the only reasonable way to systematically study *P. mendocina* infections. If studies describing less than four patients had been excluded, only one study would have been left for inclusion with only four patients with CNS infections [2].

To conclude, *P. mendocina* is a Gram-negative bacterium known to mainly cause IE, CNS infections, SSTIs, bacteremias and musculoskeletal infections. Even though ampicillin resistance is high, it shows notable sensitivity to most other antibiotics. Physicians looking after patients at risk should familiarize themselves with these infections.

## Figures and Tables

**Table 1:** Characteristics of 16 patients with infections by *Pseudomonas mendocina*: site of infection, antimicrobial resistance, treatment and outcomes. Values show cases among patients with available data.

Characteristic	Value
Male, n (%)	12 out of 16 (75%)
Age, mean (+/− SD) in years	53.5 (19.9)
**Medical History**	
HIV infection, n (%)	1 out of 16 (6.3%)
Central venous catheter, n (%)	1 out of 16 (6.3%)
Previous antibiotic use, n (%)	1 out of 16 (6.3%)
No known predisposing factors, n (%)	7 out of 16 (43.8%)
**Site of Infection**	
Infective endocarditis, n (%)	4 out of 16 (25%)
Central nervous system infection, n (%)	4 out of 16 (25%)
Skin and soft tissue infections, n (%)	3 out of 16 (18.8%)
Bacteremia (without endocarditis), n (%)	2 out of 16 (12.5%)
Musculoskeletal infections, n (%)	2 out of 16 (12.5%)
Peritoneal dialysis-associated peritonitis, n (%)	1 out of 16 (6.3%)
**Data on Infection**	
Presence of fever, n (%)	7 out of 10 (70%)
Presence of sepsis, n (%)	4 out of 9 (44.4%)
**Antimicrobial Resistance**	
Ampicillin resistance, n (%)	4 out of 5 (80%)
Co-trimoxazole resistance, n (%)	1 out of 3 (33.3%)
Carbapenem resistance, n (%)	1 out of 10 (10%)
Third or fourth generation cephalosporin resistance, n (%)	0 out of 11 (0%)
Aminoglycoside resistance, n (%)	0 out of 10 (0%)
Quinolone resistance, n (%)	0 out of 7 (0%)
Piperacillin–tazobactam resistance, n (%)	0 out of 6 (0%)
Colistin resistance, n (%)	0 out of 1 (0%)
**Treatment of *P. mendocina* IE**	
Third or fourth generation cephalosporins, n (%)	8 out of 15 (53.3%)
Quinolones, n (%)	5 out of 15 (33.3%)
Aminoglycosides, n (%)	3 out of 15 (20%)
Carbapenems, n (%)	2 out of 15 (13.3%)
Colistin, n (%)	2 out of 15 (13.3%)
Piperacillin–tazobactam, n (%)	1 out of 15 (6.7%)
Aminopenicillins, n (%)	1 out of 15 (6.7%)
Co-trimoxazole, n (%)	1 out of 15 (6.7%)
**Outcome**	
Clinical cure, n (%)	14 out of 15 (93.3%)
Deaths due to the infection, n (%)	0 out of 15 (0%)
Deaths overall, n (%)	1 out of 15 (6.7%)

SD: standard deviation; HIV: human immunodeficiency virus.

**Table 2:** Characteristics of four patients with infective endocarditis by *Pseudomonas mendocina*: antimicrobial resistance, treatment and outcomes. Values show cases among patients with available data.

Characteristic	Value
Male, n (%)	3 out of 4 (75%)
Age, mean (+/− SD) in years	46 (16.7)
**Medical History**	
Previous cardiac surgery, n (%)	2 out of 4 (50%)
Prosthetic cardiac valve, n (%)	1 out of 4 (25%)
No known predisposing factors, n (%)	3 out of 4 (75%)
Concomitant bacteremia, n (%)	4 out of 4 (100%)
**Data on Infection**	
Duration of symptoms, median (IQR) in days	7 (5–45)
Patients with fever, n (%)	4 out of 4 (100%)
Patients with sepsis, n (%)	2 out of 3 (66.7%)
**Antimicrobial Resistance**	
Ampicillin resistance, n (%)	3 out of 4 (75%)
Aminoglycoside resistance, n (%)	0 out of 4 (0%)
Third or fourth generation cephalosporin resistance, n (%)	0 out of 3 (0%)
Piperacillin–tazobactam resistance, n (%)	0 out of 3 (0%)
Quinolone resistance, n (%)	0 out of 3 (10)
Carbapenem resistance, n (%)	0 out of 2 (0%)
Co-trimoxazole resistance, n (%)	0 out of 1 (0%)
Colistin resistance, n (%)	0 out of 1 (0%)
**Treatment of *P. mendocina* Infections**	
Third or fourth generation cephalosporin, n (%)	2 out of 4 (50%)
Quinolones, n (%)	2 out of 4 (50%)
Aminoglycoside, n (%)	2 out of 4 (50%)
Piperacillin–tazobactam, n (%)	1 out of 4 (25%)
Carbapenems, n (%)	1 out of 4 (25%)
Surgical management, n (%)	2 out of 4 (50%)
Duration of treatment, median (IQR) in days	49 (42–82.3)
**Outcome**	
Clinical cure, n (%)	4 out of 4 (100%)
Deaths due to the infection, n (%)	0 out of 4 (0%)
Deaths overall, n (%)	0 out of 4 (0%)

SD: standard deviation; IQR: intraquartile range.

**Table 3:** Characteristics of four patients with central nervous system infections by *Pseudomonas mendocina*: treatment and outcomes. Values show cases among patients with available data.

Characteristic	Value
Male, n (%)	2 out of 4 (50%)
Age, mean (+/− SD) in years	69.5 (11.3)
**Medical History**	
Community acquired infection, n (%)	2 out of 4 (50%)
Hospital acquired infection, n (%)	2 out of 4 (50%)
No known predisposing factors, n (%)	2 out of 4 (50%)
**Treatment of *P. mendocina* CNS Infection**	
Third or fourth generation cephalosporins, n (%)	3 out of 4 (75%)
Carbapenems, n (%)	1 out of 4 (25%)
**Outcome**	
Clinical cure, n (%)	4 out of 4 (100%)
Deaths due to the infection, n (%)	0 out of 4 (0%)
Deaths overall, n (%)	0 out of 4 (0%)

SD: standard deviation; CNS: central nervous system.
